# Microwave‐Assisted Organic Syntheses in Deep Eutectic Solvents: A Win‐Win Association for Sustainable Chemistry

**DOI:** 10.1002/open.202500478

**Published:** 2025-11-07

**Authors:** Pierre‐Olivier Delaye, Chefikou Salami, Emilie Thiery

**Affiliations:** ^1^ Laboratoire Synthèse et Isolement de Molécules Bioactives (SIMBA, UR 7502) Université de Tours, Faculté de Pharmacie Tours France

**Keywords:** deep eutectic solvents, green chemistry, microwave heating, microwave‐assisted syntheses, sustainability

## Abstract

The 12 principles of green chemistry guide the scientific community toward the development of chemical processes that are more respectful of the environment and safer for human health. In organic synthesis, this mainly involves the use of sustainable alternatives to conventional organic solvents, energy‐efficient processes, and waste minimization. In this context, this review focuses on the use of deep eutectic solvents (DES) in microwave‐assisted organic synthesis. Indeed, DES, due to their nonvolatility, nonflammability, and low toxicity compared to conventional organic solvents, are considered desirable “green solvents” for the development of environmentally friendly processes. Moreover, their physicochemical properties make them ideal media for microwave heating. Thus, all organic syntheses using DES as solvent and microwave heating documented in the literature are reported, including heterocycle synthesis, nitrogen quaternization reactions, 5‐hydroxymethylfurfural production, Knoevenagel reactions, and miscellaneous transformations. The recyclability of DES‐based systems and their scalability, where applicable, are reported. Mechanistical considerations when DES are involved are also described. Compared with conventional heating methods, microwave heating of DES media generally results in good yields and a significant reduction in reaction times. This DES‐MW combination appears promising for more sustainable organic syntheses.

## Graphical abstract

In organic synthesis, deep eutectic solvents (DES) have demonstrated their ability to be used as reaction media for the development of reactions in line with green chemistry principles. This review presents an overview of microwave‐assisted organic synthesis in DES, highlighting the diversity of uses for these solvents, their role in mechanisms, the sustainability of methodologies, and the research and development needs in this promising field.

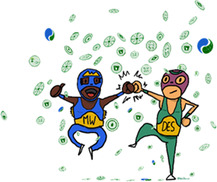

## 1 Introduction

**FIGURE 1:**
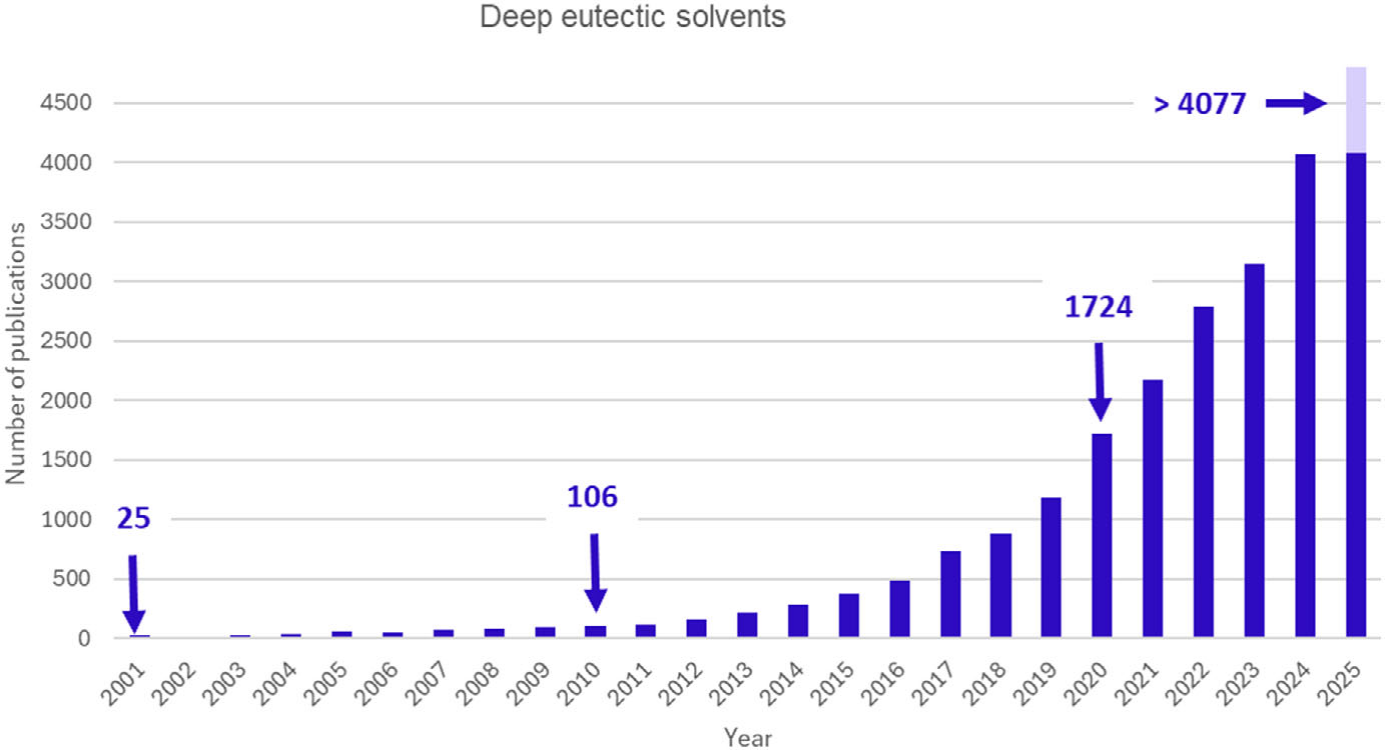
Number of publications related to DES (source SciFinder).

The 12 principles of green chemistry proposed by Anastas et al. aim to minimize the risks and environmental impact of chemical practices [1]. They guide researchers in the development of more sustainable methodologies. Reaction solvents significantly influence the environmental impact, cost, health, and safety issues of chemical processes [2]. The fifth principle states that “the use of auxiliary substances (e.g. solvents, separation agents, etc.) should be made unnecessary as far as possible and harmless when used.” Indeed, the volatile organic solvents commonly used in academic and industrial organic synthesis for their solubilizing properties are toxic and harmful to both users and environment. One of the challenges of green chemistry is the replacement of conventional organic solvents with safer, more sustainable alternatives. The scientific literature reports several alternatives, including water [3], biosolvents [4], and ionic liquids [5]. More recently, deep eutectic solvents (DES) have been considered as a promising alternative due to their nonvolatility, nonflammability, and low toxicity compared to conventional organic solvents [6, 7–8]. Since their initial description by Abbott et al. in 2001 [9], DES have been a subject of significant interest from the scientific community, as demonstrated by the steady increase in annual publications, reaching a peak of 4067 articles in 2024 (Figure 1).

**FIGURE 2:**
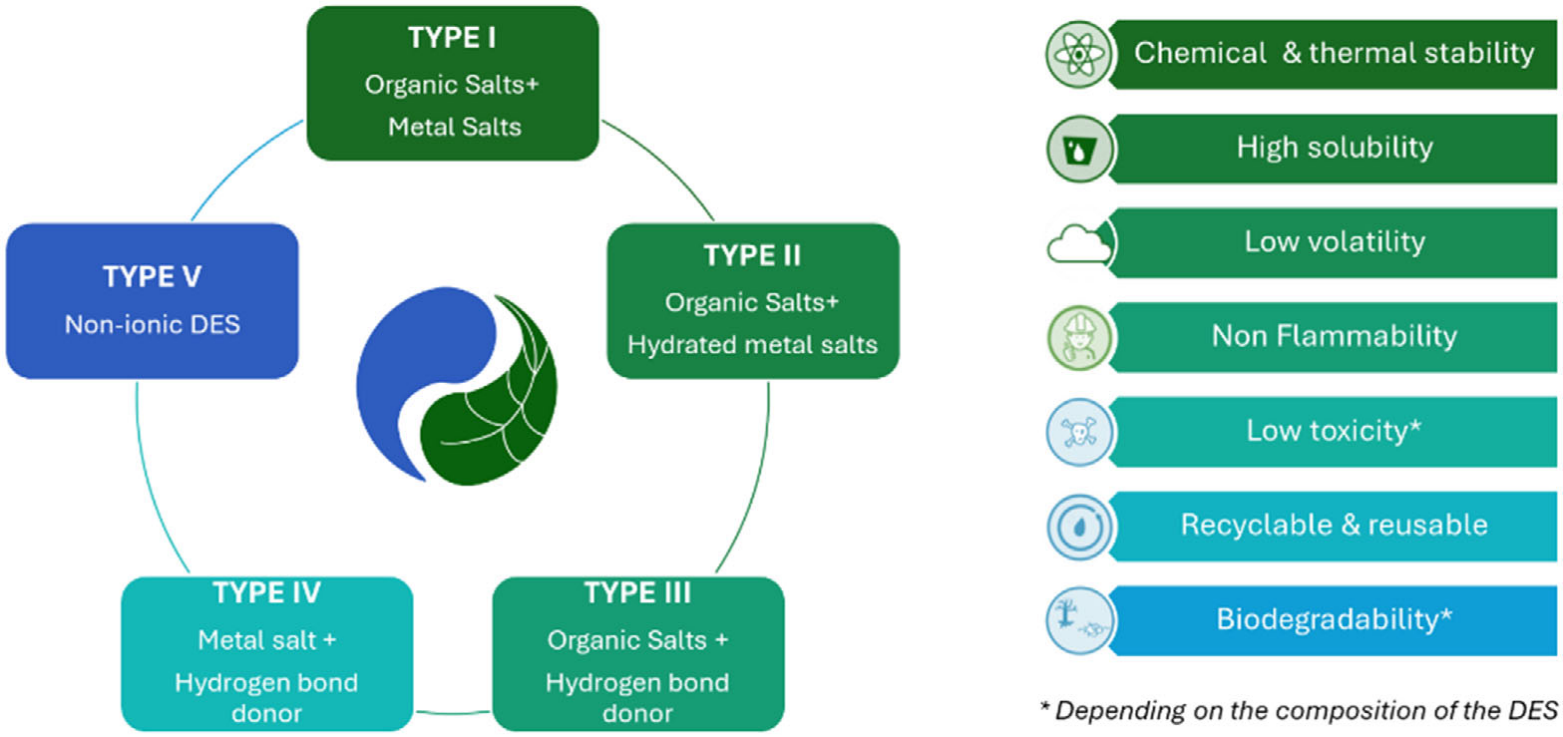
Properties and different classes of DES.

Originally defined as mixtures of Lewis/Brønsted acids and bases, the most widespread definition is based on a mixture of several compounds, including at least one hydrogen bond donor (HBD) and one hydrogen bond acceptor (HBA). More recently, some authors have proposed a broader definition based solely on thermodynamic properties, encompassing a wider range of DES based on compounds that are neither acids nor bases [10]. In DES, the decrease in melting point of the mixture compared to the melting points of its components is due to charge delocalization occurring by hydrogen bonding. Currently, there are five classes of DES, which are classified according to their composition (Figure 2). The diversity of components enables the formulation of a wide range of solvents with varied physicochemical properties [11, 12]. When the components come from natural resources, they are called NaDES (natural deep eutectic solvent). In addition to the properties described above, these solvents meet green chemistry objectives thanks to their biodegradability and durability (Figure 2) [13].

In recent decades, DES have been described as the “reaction medium of the century” [14] and have found applications in almost all areas of chemistry [6–8, 15]. For example, they are used in metabolite extraction, electrochemistry, carbon dioxide capture, and cosmetics formulation. Organic chemists are also interested in using these new solvents because of their physicochemical properties and durability [16, 17–18]. They have been used as a medium, and sometimes as a catalyst [19], for heterocycle synthesis [20], carbon–carbon coupling reactions [21], and multicomponent reactions [22].

**FIGURE 3:**
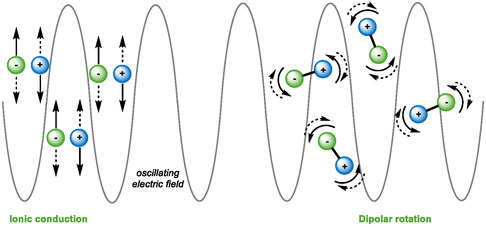
Schematic illustration of two main mechanisms of microwave heating.

To improve the fluidity and homogeneity of reaction media, they are often heated or subjected to various activation techniques. Among the various existing methods, microwave heating has been recognized as a reliable tool for organic synthesis [23]. Microwaves are electromagnetic waves with frequencies between 300 MHz and 300 GHz. Microwave heating of a medium is achieved by two main mechanisms (Figure 3): (1) dipolar rotation: molecules possessing a dipolar moment orient themselves under the influence of the electric field oscillating at a given frequency; (2) electrical or ionic conduction: under the effect of microwaves, charged molecules oscillate back and forth, colliding with neighboring molecules and generating thermal energy. Microwave heating is considered as an environmentally friendly alternative to traditional heating methods. It enables high, homogeneous temperatures to be reached in reaction media and rapid reactions to be carried out, generally leading to very good yields with energy consumption usually lower than classical oven. Since the first microwave‐assisted organic syntheses were described in 1986 [24, 25], interest in this technology has grown steadily.

**SCHEME 1:**
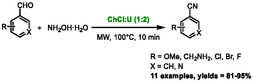
First example of microwave‐assisted organic reaction in eutectic solvent.

Eutectic solvents, composed mainly of polar and/or ionic molecules, are therefore good media for microwave heating. However, only a limited number of studies have examined the correlation between DES properties and microwave heating efficiency [26, 27–28]. Despite the lack of knowledge on this subject, DES have been widely used under microwave irradiation to improve extraction processes, particularly in the context of biomass extraction [29, 30, 31, 32–33]. It should be noted that there is a relatively limited literature on organic reactions occurring in DES under microwave heating, despite the obvious potential in this area. The first example documented in the literature was described by Patil et al. in 2013 [34]. The authors reported the single‐step synthesis of nitriles from aldehydes and hydroxylamine in a mixture of choline chloride and urea (in a 1:2 ratio) under microwave irradiation (Scheme 1). In this initial example, choline chloride:urea was identified as an effective solvent for microwave activation. It demonstrated slightly improved yields compared to classical heating and a significant reduction in reaction time, from 12 h under classical heating conditions to just 10 min through microwave irradiation.

**FIGURE 4:**
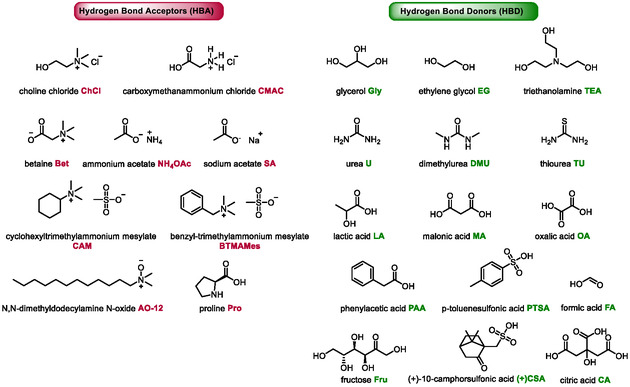
DES components used in microwave‐assisted reactions.

The objective of this review is to encompass all organic syntheses documented in the literature subsequent to this pioneering example, employing DES as a solvent and microwave heating. This includes the synthesis of heterocycles, reactions of nitrogen quaternization, the production of 5‐hydroxymethylfurfural, Knoevenagel reactions, and miscellaneous transformations. In alignment with green chemistry principles, emphasis will be placed on the recyclability of DES‐based systems and their scalability when appropriate, as well as mechanistical considerations when DES are involved. Figure 4 presents a comprehensive list of all the components used in the composition of DES mentioned in this review. All the ratios described in this review are given as mol/mol ratios.

## 2 Heterocycles Syntheses

The majority of publications dealing with microwave‐assisted organic synthesis in DES have focused on the synthesis of heterocycles. These include nitrogen‐containing, oxygen‐containing, both nitrogen‐ and oxygen‐containing, and finally, nitrogen‐ and sulfur‐containing heterocycles.

### 2.1 Nitrogen‐Containing Heterocycles

#### 2.1.1 Pyridine‐Related Structures

**SCHEME 2:**
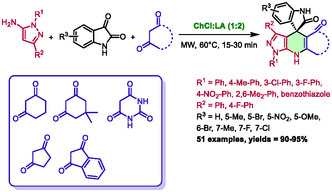
Microwave‐assisted synthesis of spiro[indoline‐3,4′‐pyrazolo[3,4‐*b*]quinolines] in choline chloride:lactic acid (1:2).

In 2019, Zhang et al. described the synthesis of spiro[indoline‐3,4′‐pyrazolo[3,4‐*b*]quinolines] in a choline chloride:lactic acid DES under microwave irradiation [35]. After a thorough screening of solvents, they found the DES choline chloride:lactic acid (1:2) to be the more suitable for the desired transformation. The reaction is a three‐component process involving pyrazol‐5‐amine, isatin, and enolizable diketones (Scheme 2). The majority of publications dealing with microwave‐assisted organic synthesis in DES have focused on the synthesis of heterocycles. These include nitrogen‐containing, oxygen‐containing, both nitrogen‐ and oxygen‐containing, and finally, nitrogen‐ and sulfur‐containing heterocycles.

**SCHEME 3:**
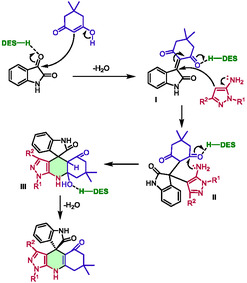
Proposed mechanism for the preparation of spiro[indoline‐3,4′‐pyrazolo[3,4‐*b*]quinolines] in choline chloride:lactic acid (1:2).

Utilizing short reaction times of 15–30 min at 60°C, they successfully obtained a wide range of 51 diversely substituted compounds, achieving excellent yields of 90%–95%. The authors also proposed a mechanism in which the eutectic solvent is believed not only to act as a solvent but also as an activator of ketones through hydrogen bonding (Scheme 3). During the initial step of Knoevenagel condensation to form intermediate **I**, the acidic property of the DES is believed to activate isatin ketone and to favor the enol form of the diketone partner. Subsequently, the 5‐amino pyrazole underwent a Michael‐type addition on the C=C bond of intermediate **I** to form adduct **II**, with again an activation of the ketone from **I**. Afterward, an intramolecular cyclization occured between the amino group and the activated ketone of adduct **II**, producing intermediate **III**. This intermediate ultimately formed spiro[indoline‐3,4′‐pyrazolo[3,4‐*b*]quinolines] through dehydration.

**SCHEME 4:**
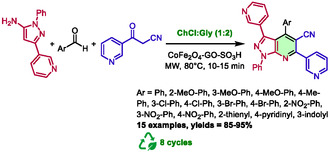
Microwave‐assisted synthesis of 3,6‐di(pyridine‐3‐yl)‐1*H*‐pyrazolo[3,4‐*b*]pyridine‐5‐carbonitriles in choline chloride:glycerol (1:2) catalyzed by CoFe_2_O_4_–GO–SO_3_H.

Previously, the same research group had developed a first synthesis of pyrazolo[3,4‐*b*]pyridines‐related structures [36]. For this study, they prepared a new magnetically separable graphene oxide anchored sulfonic acid catalyst (CoFe_2_O_4_GO–SO_3_H) and used it in choline chloride:glycerol (1:2) DES with microwave heating. The reaction also occurs through a one‐pot, three‐component process involving pyrazol‐5‐amine, but this time in combination with aldehydes and α‐cyano‐ketones. (Scheme 4).

These conditions enabled the authors to prepare 15 examples of compounds with diversely substituted phenyls and heteroaryls at the 4‐position with good to excellent yields (85%–95%) in 10–15 min at 80°C. Classical heating was found to allow the same reaction in an extended time of 120 min with a significantly lower yield (e.g., 86% versus 95% with microwave heating). It is noteworthy that both the catalyst and the DES can be recycled and reused for up to eight cycles without any substantial loss of catalytic activity. The authors also provided a plausible mechanism, similar to the one presented in Scheme 3, but without a participation from the DES.

**SCHEME 5:**
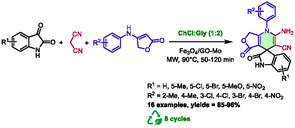
Synthesis of spiro‐oxindole dihydropyridines in choline chloride:urea (1:2) under microwave heating.

One year later, the same research group presented another magnetically separable graphene oxide‐supported catalyst, this one based on iron and molybdenum (Fe_3_O_4_/GO‐Mo), for the synthesis of spiro‐oxindole dihydropyridines from isatins, malononitrile, and anilinolactones [37]. After conducting optimization studies, they determined that the best solvent for this transformation was a mixture of choline chloride and urea (1:2), in conjunction with microwave heating at 90°C for 50–120 min (Scheme 5). It has been observed that similar yields can be obtained through classical heating methods, but with a longer reaction time (for example, 240 min). Sixteen examples with diverse isatins and anilinolactones were prepared under these conditions, with good to excellent yields (85%–96%). Notably, the catalyst can be efficiently recovered through magnetic separation and reused for up to eight cycles without a substantial decline in catalytic activity. During these cycles, the DES was also reused.

**SCHEME 6:**
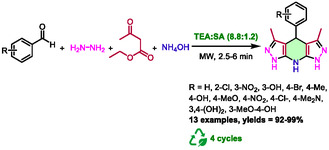
Synthesis of tetrahydropyrazolopyridines in triethanolamine:sodium acetate (8.8:1.2) eutectic solvent under microwave irradiation.

In 2021, Mirjalili et al. used the less common triethanolamine:sodium acetate (8.8:1.2) eutectic solvent for the synthesis of tetrahydrodipyrazolopyridines [38]. The reaction involves condensation of aldehydes with ethyl acetoacetate, hydrazine hydrate, and ammonium acetate in a Hantzsch‐type mechanism (Scheme 6). It is regrettable that the authors did not include the temperature used for the synthesis or the type of apparatus used in this publication.

After developing this new eutectic solvent and characterizing it by differential scanning calorimetry (DSC) and infrared (IR) spectroscopy, the authors used it to prepare 13 compounds with excellent yields of 92%–99%. Microwaves allowed an extremely short time, from 2.5 to 6 min, compared to classical heating (45 min for 40% yield). In this case, the DES serves more as a catalyst than as a solvent, given the minimal amount of 0.01 g used in the mmol‐scale reaction. It is important to note that this DES catalyst can be recycled 4 times while maintaining consistently high yields of 100%, 98%, 95%, and 92%, respectively. A mechanism has been proposed in which the DES catalyst activates carbonyl groups as hydrogen bond donors. This process is similar to the Knoevenagel condensation, Michael‐type addition, and cyclization mechanisms depicted in Scheme 3.

#### 2.1.2 Quinazoline‐Related Structures

**SCHEME 7:**
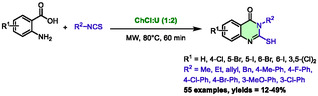
Microwave‐assisted synthesis of 2‐mercaptoquinazolin‐4(3*H*)‐ones in choline chloride:urea (1:2).

The Molnar's group presented a methodology for the synthesis of 2‐mercaptoquinazolinones in 2022 [39]. The reaction takes place between anthranilic acids and aliphatic or aromatic isothiocyanates (Scheme 7). The authors compared in this work classical heating, microwave heating, and ultrasound. In this instance, microwave irradiation proved to be the less effective method, yielding moderate results ranging from 12% to 49%. The authors proposed that urea might play a role as both a base and a hydrogen donor.

**SCHEME 8:**
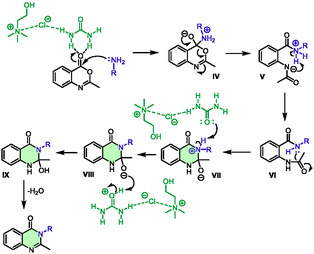
Proposed mechanism for the preparation of 2‐methyl‐3‐substituted quinazolinones in choline chloride:urea (1:2).

In 2024, the same authors published another article that completed this research [40]. They used the same methodology with benzoxazinone and anilines or hydrazides in choline chloride:urea and, again, compared different green alternatives for activating the reaction: microwave heating, ultrasound, and mechanochemical stirring. They provided 29 new examples of diversely substituted products and found that microwave heating in this case is similar to ultrasound (yields from 10% to 41% and 15% to 56%, respectively) but less efficient than mechanochemical stirring (yields from 45% to 87%). In addition, microwave heating was used on a model reaction to assess the recyclability of the system. The reaction demonstrated reasonably consistent efficiency over four cycles. The authors proposed a mechanism involving the DES in several steps. First, the amine attacked the carbonyl of the benzoxazinone, which is activated through hydrogen bonding with urea to form intermediate **IV** (Scheme 8). This intermediate subsequently underwent a ring‐opening reaction and the zwitterionic intermediate **V** evolved into **VI** through an acid/base reaction.

Then, the key step involved an intramolecular cyclization, which produced another zwitterionic intermediate **VII**. Consequently, a sequence of deprotonation of ammonium followed by protonation of alcoholate produced intermediates **VIII** and then **IX**. The carbonyl group of urea is believed to act as a shuttle for the proton. After undergoing a loss of water, quinazolinones were ultimately obtained.

**SCHEME 9:**
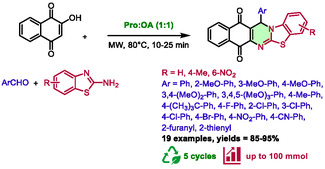
Microwave‐assisted synthesis of 13‐aryl‐13*H*‐benzo[*g*]benzothiazolo[2,3‐*b*]quinazoline‐7,12‐diones in proline:oxalic acid (1:1).

In 2017, Zhang's group reported another example of the synthesis of quinazoline‐related structures [41]. In this three‐component reaction of aromatic aldehydes, 2‐aminobenzothiazoles, and 2‐hydroxy‐1,4‐naphthoquinone, 13‐aryl‐13*H*‐benzo[*g*]benzothiazolo[2,3‐*b*]quinazoline‐7,12‐diones were obtained by using microwave irradiation of an proline:oxalic acid (1:1) eutectic mixture (Scheme 9).

**SCHEME 10:**
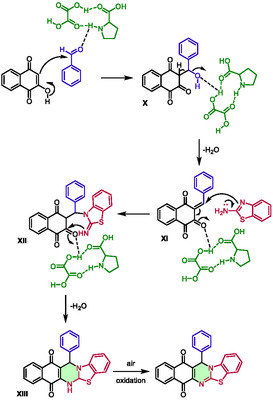
Proposed mechanism for the preparation of 13‐aryl‐13*H*‐benzo[*g*]benzothiazolo[2,3‐*b*]quinazoline‐7,12‐diones in proline:oxalic acid (1:1).

This method has been successfully employed to synthesize 19 compounds, with reaction times ranging from 10 to 25 min and temperatures of 80°C, resulting in excellent yields of 85%–95%. Microwave irradiation has been found to dramatically accelerate the reaction, with similar yields only attainable through classical heating after 120 min. Remarkably, these conditions could be applied on a larger scale (100 mmol) with an excellent yield of 94%. The recyclability of the system was also investigated, and five consecutive cycles were carried out with yields of 92%, 90%, 89%, 86%, and 85%, respectively. The eutectic mixture was described as a dual solvent/catalyst in this reaction, with the DES thought to activate carbonyls via hydrogen bonding (Scheme 10). This activation promoted the first step, a Knoevenagel condensation between 2‐hydroxy‐1,4‐naphthoquinone and aldehydes, providing intermediate **X**, and then **XI**, after the loss of a molecule of water. Subsequently, 2‐aminobenzothiazoles attacked **XI** via a Michael‐type addition and the obtained intermediate **XII** underwent intramolecular cyclization to yield intermediate **XIII**. The latter furnished 13‐aryl‐13*H*‐benzo[*g*]benzothiazolo[2,3‐*b*]quinazoline‐7,12‐diones after air oxidation.

#### 2.1.3 Quinoline‐Related Structures

**SCHEME 11:**
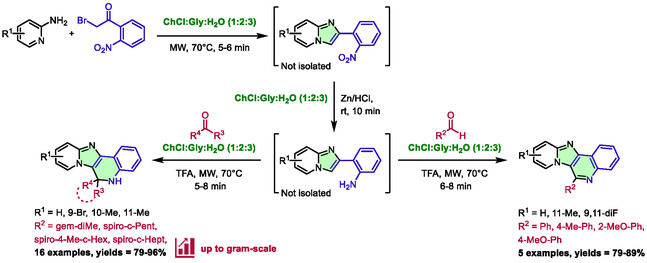
Microwave‐assisted synthesis of pyrido[2′,1′:2,3]imidazo[4,5‐*c*]quinolines and 5,6‐dihydropyrido[2′,1′:2,3]imidazo[4,5‐*c*]quinolines in choline chloride:glycerol:water (1:2:3).

Dasmahapatra et al. recently described an innovative synthesis of 5,6‐dihydropyrido [2′,1′:2,3]imidazo[4,5‐*c*]quinolines and pyrido [2′,1′:2,3]imidazo[4,5‐*c*]quinolines [42]. The synthesis was accomplished by a one‐pot sequence of three reactions, all of which used a DES as the reaction medium, and two of which used microwave heating. While optimizing each step independently, the authors found the DES choline chloride:glycerol:water (1:2:3) to be the most effective for the three reactions. Subsequently, they tried the one‐pot process, by realizing the three reactions consecutively, with the same reaction medium (Scheme 11). The first step involves synthesizing imidazo[1,2‐*a*]pyridines with a 2‐nitrophenyl group at the 2‐position. This is achieved by reacting various 2‐aminopyridines with 2′‐nitrophenylacyl bromide. After an expeditious reaction time of 5–6 min at 70°C under microwave heating, zinc dust and hydrochloric acid are directly added to reduce the nitro group into amine (second step) in 10 min at room temperature. Finally, Pictet–Spengler cyclization was used, with TFA and aldehydes or ketones added, to produce pyrido [2′,1′:2,3]imidazo[4,5‐*c*]quinolines or 5,6‐dihydropyrido [2′,1′:2,3]imidazo[4,5‐*c*]quinolines, respectively, after 5–8 min under microwave irradiation (70°C). The scope of the process is limited to acetone, cycloalkyl ketones, and aromatic aldehydes. However, the yields obtained are consistently good to excellent (79%–96%). A gram‐scale reaction was also successfully executed for the final reaction between 2‐(2‐aminophenyl)‐imidazo[1,2‐*a*]pyridine and cyclopentyl ketone. An excellent yield of 92% was obtained.

#### 2.1.4 Indole‐Related Structures

**SCHEME 12:**
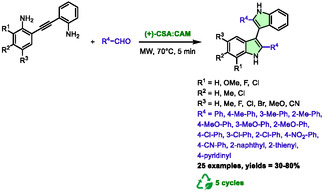
Microwave‐assisted synthesis of 3,3′‐biindoles in (+)‐camphorsulfonic acid:cyclohexyltrimethylammonium mesylate**.**

In 2023, Brambilla et al. described the use of an acidic DES as an active medium for the synthesis of 3,3′‐biindoles from 2,2′‐diaminotolane under microwave heating [43]. To this purpose, they screened 12 different DES from 14 components. Given the established knowledge that this transformation is classically acid‐catalyzed, their investigations were focused on acidic HBDs (e.g., glycolic acid, salicylic acid, or *p*‐toluenesulfonic acid). The optimal DES for the model reaction between 2,2′‐diaminotolane and tolualdehyde was determined to be a mixture of (+)‐10‐camphorsulfonic acid ((+)‐CSA) and cyclohexyltrimethylammonium mesylate (CAM) in a ratio that was not specified in the article (Scheme 12). This DES enabled the synthesis of the product within 5 min of microwave heating at 70°C, with 76% yield, demonstrating its effectiveness and efficiency in chemical synthesis processes.

**SCHEME 13:**
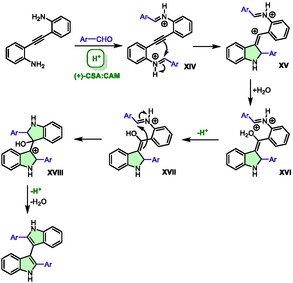
Proposed mechanism for the preparation of 3,3′‐biindoles in (+)‐camphorsulfonic acid:cyclohexyltrimethylammonium mesylate.

These conditions proved to be quite general, as 25 examples were obtained from variously substituted reagents. The yields ranged from 30% to 80%. The aldehyde partners can be substituted with a variety of phenyls and heteroaryls, including thiophene and pyridine. It was observed that the only aliphatic aldehyde did not result in the formation of any product. This system can be recycled up to 5 times with relatively consistent yields, ranging from 72% to 60%. The proposed mechanism involved an acid activation of the aldehyde to form iminium ion **XIV** (Scheme 13), which then underwent a 5‐*endo*‐*trig* aza‐Prins cyclization to form carbocation **XV**. The latter is quenched by water, producing enol **XVII** through **XVI**. The process of generation of **XVIII** from **XVII** occurs through a nucleophilic attack from the double bond to the remaining iminium. The desired biindole is finally produced by the loss of a water molecule and a proton.

#### 2.1.5 Pyrrolidone‐Related Structures

**SCHEME 14:**
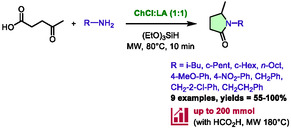
Microwave‐assisted synthesis of pyrrolidones in choline chloride:lactic acid (1:1).

Tabasso and co‐workers recently developed a sustainable route to *N*‐substituted pyrrolidones using DES and microwave heating [44]. The method is based on the reductive amination of levulinic acid, which leads to pyrrolidinones after cyclization. Various H‐donors were tested for their ability to reduce the imine, including ammonium formate, formic acid, and triethoxysilane. All reagents yielded satisfactory product yields. However, triethoxysilane was found to be more efficient, with the reaction proceeding at lower temperatures (80°C instead of 180°C for ammonium formate and formic acid) in the choline chloride:lactic acid DES (Scheme 14). The scope of the study included amines with substituted phenyl and benzyl groups, as well as aliphatic and cyclic alkyls. The results showed that the yields ranged from moderate to excellent (55%–100%) and were achieved within 10 min. During this reaction, the DES is believed to act as a multifunctional reagent, activating triethoxysilane and catalyzing the cyclization. Notably, the conditions involving formic acid at 180°C were successfully scaled up to 20 and 200 mmol, yielding 99% and 93%, respectively.

### 2.2 Oxygen‐Containing Heterocycles

**SCHEME 15:**
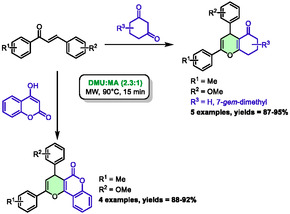
Microwave‐assisted synthesis of pyranochromenes and benzopyrans in dimethyl urea:malonic acid (2.3:1).

In 2022, Teja et al. employed 4‐hydroxycoumarin and substituted 1,3‐cyclohexanediones as nucleophiles with chalcones to promote the synthesis of pyranochromenes or benzopyrans, respectively, in DES under microwave irradiation [45]. To this end, they identified dimethylurea (DMU):malonic acid (MA) (2.3:1) as the optimal solvent for this method. Microwave heating at 90°C enabled a brief reaction time of 15 min (Scheme 15). Diversely substituted substrates were used to prepare five examples of benzopyrans and four examples of pyranochromenes with good to excellent yields (87%–95%).

In addition to the aforementioned chalcones, other derivatives were utilized, including quinoline‐derivated chalcones and dibenzylidenecyclohexane‐chalcones. These substrates resulted in the generation of novel compounds, often yielding products that were not anticipated. For instance, quinoline‐derivated chalcones, when reacted with 1,3‐cyclohexanediones, produced unexpected tetrahydrobenzofurans.

**SCHEME 16:**
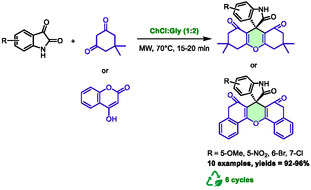
Microwave‐assisted synthesis of pyrane‐related structures in choline chloride:glycerol (1:2).

Another publication by Zhang et al. utilized the same nucleophiles (4‐hydroxycoumarin and substituted 1,3‐cyclohexanediones), but with isatins derivatives, to promote the synthesis of pyrane‐related heterocycles [46]. These reactions were carried out in a mixture of choline chloride and glycerol (in a molar ratio of 1:2) using microwave heating at 70°C for 15–20 min. Ten examples were prepared using these conditions, resulting in excellent yields ranging from 92% to 96% (Scheme 16). Classical heating led to only 82% yield after 90 min. The system has demonstrated a high degree of reusability. It has successfully been reused six consecutive times without substantial loss of yield. The authors proposed a mechanism in which the DES activated the carbonyls during a sequence of Knoevenagel condensation and Michael‐type addition. This mechanism is very similar to the one proposed in a previous paper (Scheme 3).

**SCHEME 17:**
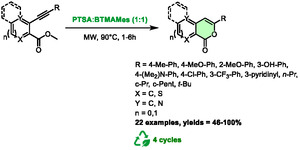
Microwave‐assisted synthesis of pyranones in *p*‐toluenesulfonic acid:benzyl‐trimethylammonium mesylate (1:1).

In 2019, the Abbiati's group published an elegant synthetic method for producing pyranones *via* 6‐*endo*‐*dig* cyclization of 2‐alkynyl‐arylcarboxylates [47]. Rather than employing a metal catalyst, which is frequently utilized in such transformations, they capitalized on the acidity of PTSA‐based DES to enhance the reaction. After conducting optimization studies, it was determined that *p*‐toluenesulfonic acid:benzyl‐trimethylammonium mesylate (BTMAMes) was the most effective DES when subjected to microwave heating at a temperature of 90°C for a range of 1–6 h. Heating with an oil bath required 22–29 h to obtained comparable yields. The scope is quite wide, as the alkyne can be substituted by aliphatic and cyclic alkyls, heterocycles, and electron‐rich phenyls (Scheme 17).

When the alkyne was substituted with an electron‐withdrawing group, the authors obtained benzofuranones as the main product from *5‐exo‐dig* cyclization. The regioselectivity can then be tuned by adding a catalytic amount of silver(I) salt to obtain the pyranone. In addition, the system without silver was recycled over 4 runs with excellent yields (92‐98%) on the model reaction.

### 2.3 Nitrogen‐ and Oxygen‐Containing Heterocycles

#### 2.3.1 Benzoxazole‐Related Structures

**SCHEME 18:**
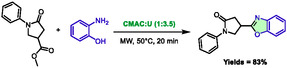
Microwave‐assisted synthesis of benzoxazole in carboxymethanammonium chloride:urea (1:3.5).

In 2021, Riadi et al. reported the synthesis of benzoxazole from esters and aminophenol in a DES composed of carboxymethanammonium chloride (CMAC) and urea (1:3.5) [48]. Although the work focused more on ultrasound activation, the authors also evaluated microwave heating during optimization, which resulted in similar yields: 83% versus 88% for ultrasound (Scheme 18).

**SCHEME 19:**
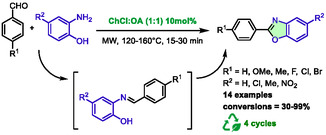
Microwave‐assisted synthesis of benzoxazoles using choline chloride:oxalic acid (1:1) as catalyst.

A year later, Pham and her colleagues presented a method for the preparation of benzoxazoles from aldehydes and aminophenols using a catalytic amount of choline chloride:oxalic acid (1:1) under microwave heating [49]. In this study, DES was utilized as a catalyst rather than a solvent, and the optimal conditions were identified using 10 mol% of DES (Scheme 19). Classical heating, when associated with comparable or extended reaction times (60 min), has been shown to result in very low conversion rates of 14% and 25%, respectively.

Unfortunately, depending on the substituents on both aldehydes and aminophenols, the authors often obtained a mixture of the desired product and the intermediate imine. They also note that the conversion is strongly dependent on the aminophenol substituents, as the 2‐amino‐4‐nitrophenol ended with low conversions (30%–42%). A proposed mechanism for this reaction involves the use of DES as both a base and an acid, activating the carbonyl to promote the formation of the imine intermediate, followed by the attack of phenol to form the cycle. The reuse of the catalytic system was also studied on the model reaction between 2‐amino‐4‐chlorophenol and benzaldehyde. Recycling was carried out for four runs, resulting in a slight decrease in conversion and an increased imine/product ratio.

### 2.4 Nitrogen and Sulfur‐Containing Heterocycles

#### 2.4.1 Thiadiazolines‐Related Structures

**SCHEME 20:**
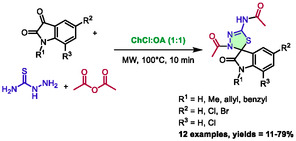
Microwave‐assisted synthesis of spiro‐1,3,4‐thiadiazolines in choline chloride:oxalic acid (1:1).

In 2021, Castro et al. described a one‐pot, multicomponent synthesis of spiro‐1,3,4‐thiadiazolines in DES under microwave irradiation [50]. The reaction took place between isatin derivatives, thiosemicarbazide, and acetic anhydride. During the optimization process, it was determined that a mixture of choline chloride and oxalic acid at a ratio of 1:1 (DES) was the most effective solvent at a temperature of 100°C, with a reaction time of 10 min. This methodology was applied to a series of diversely substituted isatin derivatives to synthesize 12 examples with yields ranging from poor to good (11%–79%) (Scheme 20). Additionally, the biological activity of the compounds was assessed against cancer cells lines. Some of the best molecules exhibited interesting IC_50_ against small cell lung cancer cells (3.53 µM) and promyelocytic leukemia (3.28 µM).

**SCHEME 21:**
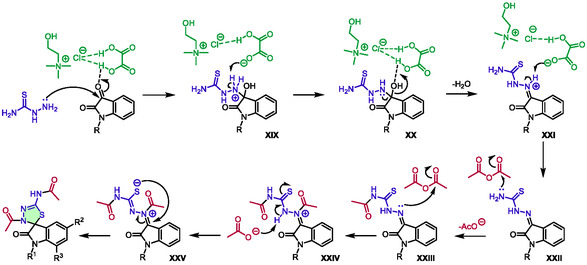
Proposed mechanism for the synthesis of spiro‐1,3,4‐thiadiazolines in choline chloride:oxalic acid (1:1).

The authors also compared this methodology with one of their previous publications, in which they described the same synthesis but in a multistep process [51]. Interestingly, for some substrates, the one‐pot process using DES gives better yields than multistep synthesis (e.g., 79% and 46%, respectively, for *N*‐methyl isatin). A proposed mechanism involved the eutectic solvent functioning as both an acid and a base (Scheme 21). This activated the carbonyl group from isatin, enabling the attack of thiosemicarbazide to form **XIX**. Therefore, a sequence of deprotonation, dehydration, and deprotonation leads to **XXII** through **XXI**. Subsequently, intermediate **XXII** underwent a double acetylation on two of its nitrogen atoms (intermediates **XXIII** and then **XXIV**). Finally, intermediate **XXV** formed spiro‐1,3,4‐thiadiazolines via intramolecular cyclization of the sulfur on iminium.

#### 2.4.2 Thiazolidines‐Related Structures

**SCHEME 22:**
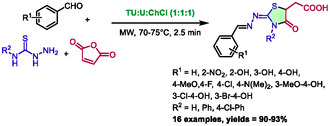
Microwave‐assisted synthesis of 4‐thiazolidinone‐5‐carboxylic acid in thiourea:urea:choline chloride (1:1:1).

In 2022, Shaikh and co‐workers developed a method for synthetizing 4‐thiazolidinone‐5‐carboxylic acid from aldehydes, thiosemicarbazides, and maleic anhydride in a thiourea:urea:choline chloride (1:1:1) deep eutectic mixture [52]. This method uses microwave heating, which enables a remarkably brief reaction time of 2.5 min at 70°C–75°C. A library of 16 compounds was obtained with excellent yields (90%–93%) (Scheme 22).

## 3 Nitrogen Quaternization

**SCHEME 23:**
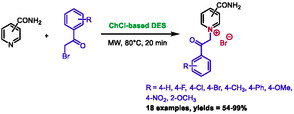
Microwave‐assisted synthesis of phenylacyl pyridinium compounds in choline chloride‐based DES.

The association between DES and microwave heating has also been documented in the literature to promote nitrogen quaternization. The initial examples were provided by Bušić and colleagues in 2020 and 2022 [53, 54]. They described the synthesis of pyridinium salts by reacting isonicotinamide or nicotinamide with substituted 2‐bromoacetophenone in DES under different conditions. The study compared classical heating, ultrasound, and microwave irradiation. In both publications, microwave heating was identified as the optimal method, demonstrating enhanced yields and reduced reaction times (e.g., 26% in 4 h at 80°C for classical heating; 34% in 3 h at 80°C for ultrasound; and 96% in 20 min at 80°C for microwave heating). The authors used a choline chloride‐based DES, with choline chloride:levulinic acid (1:2), choline chloride:oxalic acid (1:1), and choline chloride:urea (1:2) giving the best results. Eighteen products were prepared by this method, yielding results ranging from 54% to 99% (Scheme 23).

**SCHEME 24:**
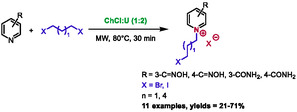
Microwave‐assisted synthesis of haloalkyl pyridinium salts in choline chloride:urea (1:2).

Recently, the same research group presented an extension of this methodology for the reaction of pyridines and dihaloalkanes [55]. For this study, choline chloride:urea (1:2) was chosen as solvent and classical heating and microwave irradiation were also compared. It was surprising to find that DES was not the most effective solvent for this synthesis, as acetone yielded significantly higher results (32%–98% for acetone and 21%–71% for DES) (Scheme 24). However, it is crucial to consider the disparity in sustainability between acetone and DES when assessing these differences in yields. These pyridinium salts were also tested for their antifungal activities. The inhibition rate at 10 µg/mL reached 90% for the best compound against *Sclerotinia sclerotiorum*.

## 4 Synthesis of 5‐Hydroxymethylfurfural

In the numerous publications that address microwave heating in DES, several focus on the preparation of 5‐hydroxymethylfurfural (HMF) from renewable sources, such as fructose. HMF is a chemical derived from biomass that can be converted into a variety of valuable chemicals, including furan derivatives and solvents [56].

**SCHEME 25:**
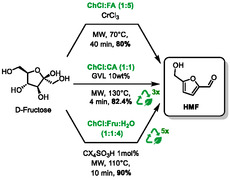
Microwave‐assisted synthesis of HMF from fructose in choline chloride:formic acid (1:5), choline chloride:citric acid (1:1), and choline chloride:fructose (1:1).

In 2020, the research group of Hu presented a synthetic route to produce HMF by dehydration of fructose [57]. Given the prevalence of acidic and/or metal chloride catalysts in such transformations, the authors opted to utilize an acidic DES composed of choline chloride and formic acid, with catalytic amounts of chromium chloride (choline chloride:formic acid (1:5) and CrCl_3_:ChCl/formic acid (1:40)) (Scheme 25, top arrow). Microwave heating of the reaction resulted in increased yields of approximately 15%–20%, compare to classical heating, for reaction times ranging from 10 to 40 min at 70°C (e.g., approximately 80% for microwave heating and approximately 60% for classical heating). The catalytic system was successfully recycled during five runs. However, it should be noted that the recycling process was not assessed using microwave irradiation; it was conducted using conventional heating methods.

In 2022, Morais et al. described another synthetic method to prepare HMF from fructose [58]. Following a thorough screening study, it was determined that a system composed of choline chloride and citric acid at a molar ratio of 1:1 is optimal. This system was completed by γ‐valerolactone (GVL) to decrease the viscosity. Furthermore, the authors optimized all the parameters by using a design of experiments software. Response surface methodology (RSM) was utilized to establish the optimal conditions, which included microwave heating at 130°C for 4 min, 10 w% of GVL, and a solid/liquid ratio of 0.05 (Scheme 25, middle arrow). These conditions enabled the synthesis of HMF from fructose with an 82.4% yield. In addition, the recyclability was assessed over three runs, yielding satisfactory results with an average yield of 84.8% ± 1.2%.

In 2023, Castro and co‐workers presented an alternative approach to synthesizing HMF from fructose [59]. In this approach, fructose is used both as a reagent and as one of the components of the DES (HBD). The DES consists of choline chloride, fructose, and water (in a 1:1:4 ratio). The reaction utilized 1 mol% of *p*‐sulfonic acid calix[4]arene (CX_4_SO_3_H) as the acid catalyst. This effective process resulted in a high yield of HMF (90%) at 110°C in just 10 min of microwave heating (Scheme 25, bottom arrow). It is also possible for this system to be reused; however, a gradual drop‐in activity was observed over five runs, from 90% to 39%.

**SCHEME 26:**
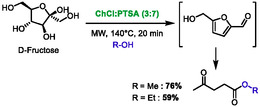
Microwave‐assisted conversion of D‐Fructose to methyl and ethyl levulinate in choline chloride:*p*‐toluenesulfonic acid (3:7).

Aslanlı and Sert reported in 2025 another synthesis of HMF from fructose, combined with its one‐pot conversion to alkyl levulinates [60]. In this study, the most efficient acidic DES was found to be a mixture of choline chloride and *p*‐toluenesulfonic acid (PTSA) in a ratio of 3:7. The reaction was conducted at 140°C under microwaves for 20 min, in the presence of either ethanol or methanol, which promote the conversion of HMF into ethyl or methyl levulinate, in 59% and 76% yield, respectively (Scheme 26). The authors compared their results to existing literature and demonstrated the efficacy of this methodology in terms of yield and reaction time.

## 5 Knoevenagel Reactions

**SCHEME 27:**
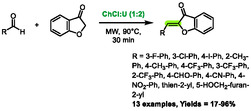
Microwave‐assisted synthesis of aurones in choline chloride:urea (1:2).

In 2017, Taylor and co‐workers developed a method to prepare aurones by combining DES and microwaves [61]. Choline chloride:urea in a 1:2 molar ratio was utilized as solvent and the reaction between benzofuran‐3‐one and various aldehydes was investigated. Microwaves consistently yielded higher results and reduced reaction time (from 2 to 13 h for classical heating to 30 min with microwaves). The only exception was 4‐nitrobenzaldehyde, which exhibited a lower yield when subjected to microwave irradiation (Scheme 27).

**SCHEME 28:**
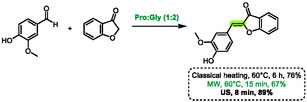
Comparison of classical heating, microwave heating and ultrasounds for the synthesis of aurones in proline:glycerol (1:2).

A few years later, the research group of Detsi also used eutectic solvents for the synthesis of aurones [62]. In their study, the authors compared classical heating, microwave heating, and ultrasound irradiation during optimization on a model reaction between benzofuran‐3‐one and vanillin. In the best DES found (proline:glycerol (1:2)), microwaves proved to be the least effective heating method, achieving 67% in 15 min (Scheme 28). This was in comparison with classical heating, which resulted in 76% in 6 h, and ultrasounds, which reached 89% in 8 min. Subsequently, the authors chose to focus the rest of the article on ultrasounds, but the findings are also relevant to microwave heating. The latter provided a satisfactory yield in only 15 min, compared to the 6 h required by classical heating.

**SCHEME 29:**
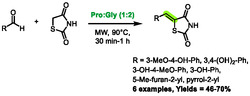
Microwave‐assisted synthesis of 5‐benzylidenethiazolidine‐2,4‐dione derivatives in proline:glycerol (1:2).

More recently, Hesse et al. developed a method to promote Knoevenagel reactions in L‐proline‐based DES [63]. The aim of this method was to synthesize arylidene rhodanines, thiazolidine‐2,4‐diones (TZD), and barbituric derivatives. If both rhodanine and barbituric derivatives proved to be reactive enough to undergo addition to aldehydes under classical heating at 60°C, the authors found TZD to be less reactive and only poor yields were obtained with these nucleophiles. Following optimization, microwave heating at 90°C was utilized to successfully overcome this limitation and yield the desired 5‐benzylidenethiazolidine‐2,4‐dione derivatives (Scheme 29). These conditions enabled shorter reaction times (e.g., 24 h for classical heating and 1 h for microwave heating) and improved yields. Six examples were prepared using this protocol, yielding results ranging from 46% to 70%. The authors proposed that the DES plays a role in the reaction mechanism, activating the carbonyl from the aldehyde via hydrogen‐bonding. Notably, this method utilizes no volatile organic compounds (VOCs) throughout the process, as the work‐up consists solely of adding water and filtration of the precipitated product.

## 6 Miscellaneous Reactions

### 6.1 Henry Reaction

**SCHEME 30:**
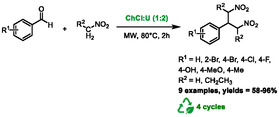
Microwave‐assisted synthesis of dinitroalkanes derivatives in choline chloride:urea (1:2).

In 2020, Colombo Dugoni et al*.* reported a method to prepare 1,3‐dinitropropanes derivatives by mixing aldehydes and nitromethane in choline chloride:urea (1:2) under microwave irradiation [64]. The authors initially sought to synthesize classical Henry products (β‐nitro alcohols), but serendipitously discovered a highly efficient and selective method to synthesize 1,3‐dinitropropanes (Scheme 30). It was discovered that heating nitromethane and 4‐fluorobenzaldehyde in the previously mentioned DES at 80°C provided an excellent yield of 96% of the 1,3‐dinitropropane product. This product was obtained through a tandem Henry/Michael addition sequence, in which the β‐nitro alcohol underwent a dehydration process to produce β‐nitrostyrene. This compound then reacted with another equivalent of nitromethane, resulting in the formation of 1,3‐dinitropropane. During the optimization process, it was determined that the use of microwave technology significantly reduced the reaction time from 24 h (conventional heating) to just 2 h. A variety of aldehydes were tested with nitromethane, yielding the desired products with satisfactory to excellent yields. Nitropropane was also utilized in the presence of 4‐fluorobenzaldehyde, resulting in the formation of the corresponding dinitro compound (88%). It is noteworthy that ketones presented more significant challenges as substrates, as only the activated trifluoromethylketone reacted with nitromethane. Contrary to the observations made with aldehydes, only the β‐nitro alcohols were obtained. Additionally, the feasibility of recycling the DES was studied. The DES was reused for four cycles, resulting in a slight decrease in yield from 100% to 84%. The authors proposed a mechanism in which urea from the DES activates the aldehyde and stabilizes the nitronate ion through hydrogen bonding.

**SCHEME 31:**
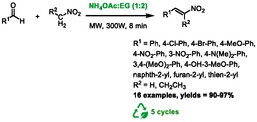
Microwave‐assisted synthesis of *trans*‐β‐nitrostyrenes in ammonium acetate:ethylene glycol (1:2).

One year later, Shaibuna and co‐workers presented an alternative method combining DES and microwave heating to promote Henry reaction [65]. The authors utilized a mixture of ammonium acetate and ethylene glycol at a 1:2 molar ratio as DES. Following the optimization of a model reaction between benzaldehyde and nitromethane, it was determined that the most efficient conditions involved the use of 1 millimole of DES per millimole of substrate, in conjunction with microwave heating at 300 W for a duration of 8 min. It should be noted that the specific temperature reached during the heating process was not specified in the report. In these conditions, the product obtained was *trans*‐β‐nitrostyrene, which was formed through the dehydration of the β‐nitro alcohol intermediate (Scheme 31). These conditions were applied to synthesize a series of various *trans*‐β‐nitrostyrenes from reaction between diversely substituted aromatic or heteroaromatic aldehydes and nitromethane or nitropropane. Excellent yields were obtained in all cases, ranging from 90% to 97%. The DES was also reused during five cycles, with a limited drop‐in activity, from 95% to 82%.

### 6.2 Propargylamines Synthesis

**SCHEME 32:**
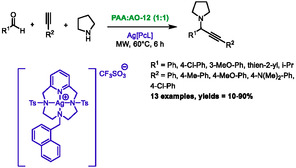
Microwave‐assisted synthesis of propargylamines synthesis in phenylacetic acid:*N*,*N*‐dimethyldodecylamine *N*‐oxide (1:1).

In 2022, the research group of Abbiati disclosed a new silver‐catalyzed aldehyde‐amine‐alkyne multicomponent reaction (A^3^‐coupling MCR) in acidic DES under microwave irradiation [66]. After conducting a thorough optimization study that included DES components, catalyst, temperature, reaction time, and heating method, it was established that the optimal conditions consist of an eutectic mixture composed of phenylacetic acid (PAA) and *N*,*N*‐dimethyldodecylamine *N*‐oxide (AO‐12) at a molar ratio of 1:1, in addition to microwave heating at 60°C for 6 h with a silver complex. The catalyst is a specific silver complex with pyridine‐containing ligands (Scheme 32). During the optimization process, the authors found that the microwave allowed for a reduction in both temperature and reaction time compared to classical heating methods. The optimized conditions enabled the model reaction between phenylacetylene, benzaldehyde, and pyrrolidine to proceed with a yield of 53%. With these conditions in hand, the authors assessed the scope and limitations for the alkyne, the aldehyde, and the amine. The alkyne partner is limited to aromatic alkynes, with electron‐rich alkynes yielding better results (80%–90%) than electron‐poor alkynes (31% for 4‐chlorophenyl and no reaction for 4‐trifluoromethylphenyl). Regarding the aldehyde partner, both aromatic and aliphatic compounds are tolerated, yielding an average to good percentage of product (44%–72%), while heteroaromatic compounds presented more of a challenge, yielding only 30% for 2‐thiophenecarboxaldehyde after 12 h. In the final analysis, the amine partner emerged as the most critical, with only low yields obtained with other amines.

### 6.3 Hydrazones Synthesis

**SCHEME 33:**
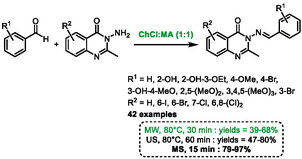
Comparison of microwave heating, ultrasounds (US) and mechanochemical synthesis (MS) for the preparation of quinazolinone‐derived hydrazone in choline chloride:malonic acid (1:2).

In 2023, Molnar et al*.* described a pathway for synthesizing quinazolinone‐derived hydrazones by reacting 3‐aminoquinazolinones with aldehydes in DES [67]. To this end, the authors used choline chloride:malonic acid (1:1) as an eutectic solvent and compared three green synthetic techniques: microwaves, ultrasounds, and a mechanochemical method, in addition to classical heating (Scheme 33). They used these techniques to synthesize 42 examples from various aldehydes and 3‐aminoquinazolinones. The results obtained from the study indicate that the mechanochemical method is the most efficient method in this case and that microwave heating is the least efficient method. Surprisingly, classical heating was found to be more effective than microwaves for this specific reaction, although the differences in yields are debatable in relation to the differences in power consumption between an oil bath and a microwave oven. As with other studies previously described, DES was proposed to participate in the mechanism by activating aldehyde via hydrogen‐bonding and deprotonating ammonium intermediates.

### 6.4 Olanzapine Derivatives Synthesis

**SCHEME 34:**
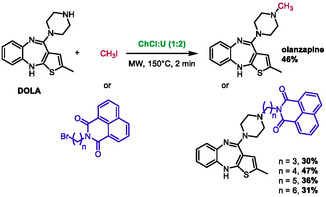
Microwave‐assisted synthesis of olanzapine and derivatives in choline chloride:urea (1:2).

In 2023, Drabczyk and co‐workers presented a synthesis of antipsychotic olanzapine and derivatives by *N*‐alkylation of DOLA (2‐methyl‐4‐piperazin‐1‐yl‐10*H*‐thieno[2,3‐*b* [1,5] benzodiazepine) under both ultrasounds and microwave irradiation (Scheme 34) [68]. This study involved the testing of various solvents and catalysts. After testing all the systems, it was determined that DMF was the most efficient. This was found to be the case when it was associated with K_2_CO_3_ and tetrabutylammonium bromide (TBAB) under microwave heating. Despite the lower yields (30%–47%) obtained when using DES (choline chloride:urea, 1:2), the authors concluded that this solvent is an interesting synthesis option due to the nontoxic nature of the DES, especially when compared to DMF. In addition, another solvent system was assessed with the addition of glycerol into the DES. However, the proportion of each component is not described in the publication. Glycerol is a well‐known component of DES, which complicates the assessment of the nature of this new mixture.

### 6.5 Suzuki‐Miyaura Cross‐Coupling

**SCHEME 35:**
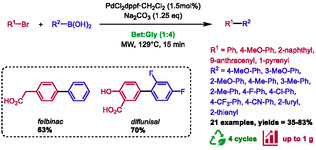
Microwave‐assisted Suzuki–Miyaura cross‐coupling reaction in betaine:glycerol (1:4).

Very recently, our team presented the first Suzuki‐Miyaura cross‐coupling reaction combining microwave heating and DES as solvent [69]. Simple conditions using PdCl_2_dppf·CH_2_Cl_2_, sodium carbonate, and betaine:glycerol (1:4) as solvent, without any ligand, were applied to the synthesis of diversely substituted biphenyls by microwave heating at 129°C for a short reaction time of 15 min (Scheme 35). The optimized conditions were obtained through the application of DoE (design of experiment). These conditions proved to be general, as both electron‐donating (OMe, Me) and electron‐withdrawing (F, CF_3_, CN) groups were successfully used for the boronic acid partner (yields ranging from 52% to 83%). In contrast, heterocycles (2‐furyl and 2‐thienyl) yielded moderate results (35%–37%). Regarding the halogenated partner, more challenging substates were used in addition to phenyl derivatives (2‐naphthyl, 77%; 9‐anthracenyl, 65%; and 1‐pyrenyl, 50%). Furthermore, two compounds of pharmaceutical interest were successfully prepared using this method. Felbinac (NSAID) and diflunisal (an analgesic and anti‐inflammatory drug) were obtained with 63% and 70% yields, respectively. Notably, the catalytic system demonstrated a capacity for recycling up to four runs, exhibiting a gradual decline in activity (75%, 80%, 74%, and 55%, respectively). A gram‐scale reaction was also performed, yielding a result of 66% compared to 75% for the mmol scale. Finally, the CHEM21 toolkit was used to assess the sustainability of this method. In order to highlight the benefits of recycling, the PMI values for the single‐cycle and four‐cycle reactions were calculated (594, 215 g.g^−^^1^, respectively), showing a reduction in the mass intensity of the process by a factor of 2.8.

## 7 Summary and Outlook

In view of their nonvolatility, nonflammability, chemical stability, and low toxicity, DES are desirable for many uses as “green solvents.” Since their first use in organic synthesis, they have demonstrated their ability to replace conventional organic solvents, particularly in ecological terms. Their polarity and often ionic nature make them ideal media for activation and heating under microwave irradiation.

The main application of DES for microwave‐assisted organic synthesis is the synthesis of nitrogen‐, oxygen‐ and/or sulfur‐containing heterocycles. Syntheses of pyrazolo‐pyridine, quinazoline, quinoline, indole, pyrrolidone, pyran, benzoxazole, thiadiazoline, and thiazolidine structures have been reported. The combined use of DES and microwaves has also enabled the quaternization of nitrogen for the preparation of pyridinium salts. A number of studies have focused on the transformation of biomass‐derived fructose or glucose for the preparation of 5‐hydroxymethylfurfural (HMF), a precursor to a variety of valuable chemicals. In addition, a number of diverse reactions were carried out using DES and microwaves, including Knoevenagel reactions, Henry reactions, propargylamine synthesis, hydrazone synthesis, synthesis of olanzapine and its derivatives, and Suzuki–Miyaura cross‐coupling.

In most cases, studies show that the combination of DES and microwaves improves yields and considerably reduces reaction times compared to traditional heating methods. Indeed, reactions often take place in less than an hour, and sometimes in just a few minutes. This enables the reduction of the process's energy consumption, which is highly desirable for green chemistry.

In a number of studies, the DES is also believed to play a role in the reaction mechanism, often by activating carbonyls through hydrogen bonding. Some mechanisms involve them as Brønsted acids or bases. To our knowledge, no mechanistic studies have yet been carried out to observe these proton migrations between DES and reaction intermediates. They would be invaluable in understanding and rationalizing the use of these solvents.

One of the advantages of DES is that it can be easily recycled, along with the reagents and catalysts it contains. Microwave heating of these media in no way alters their ability to be recycled, thus reducing reaction waste.

Some scale‐ups have been described for the synthesis of quinazoline, quinoline, pyrrolidone, or biphenyl compounds. At the scale of research laboratories, it is more difficult to work at the gram scale or above, as equipment is not always suitable. These results are encouraging and promising for the use of these reactions on a larger scale.

Given the importance attached by the chemical community to environmentally friendly processes and syntheses, the synergy between “green solvents” and energy‐saving techniques, such as microwave heating, holds great promise for the future. In this sense, the evaluation of reaction processes for quantitative and qualitative criteria such as CHEM21 [70] or DOZN [71] would enable these methodologies to be better placed in the panels of sustainable reactions.

## Conflicts of Interest

The authors declare no conflicts of interest.

## Data Availability

The data that support the findings of this study are available from the corresponding author upon reasonable request.

## References

[1] P. T. Anastas , M. M. Kirchhoff , and T. C. Williamson , “Catalysis as a Foundational Pillar of Green Chemistry,” Applied Catalysis, A: General 221 (2001): 3–13.

[2] C. Capello , U. Fischer , and K. Hungerbühler , “What Is a Green Solvent? A Comprehensive Framework for the Environmental Assessment of Solvents,” Green Chemistry 9 (2007): 927.

[3] M. Cortes‐Clerget , J. Yu , J. R. A. Kincaid , P. Walde , F. Gallou , and B. H. Lipshutz , “Water as the Reaction Medium in Organic Chemistry: From Our Worst Enemy to Our Best Friend,” Chemical Science 12 (2021): 4237–4266. 34163692 10.1039/d0sc06000c PMC8179471

[4] R. Bijoy , P. Agarwala , L. Roy , and B. N. Thorat , “Unconventional Ethereal Solvents in Organic Chemistry: A Perspective on Applications of 2‐Methyltetrahydrofuran, Cyclopentyl Methyl Ether, and 4‐Methyltetrahydropyran,” Organic Process Research & Development 26 (2022): 480–492.

[5] J. Dupont , B. C. Leal , P. Lozano , A. L. Monteiro , P. Migowski , and J. D. Scholten , “Ionic Liquids in Metal, Photo‐, Electro‐, and (Bio) Catalysis,” Chemical Reviews 124 (2024): 5227–5420. 38661578 10.1021/acs.chemrev.3c00379

[6] E. L. Smith , A. P. Abbott , and K. S. Ryder , “Deep Eutectic Solvents (DESs) and Their Applications,” Chemical Reviews 114 (2014): 11060–11082. 25300631 10.1021/cr300162p

[7] Y. Liu , J. B. Friesen , J. B. McAlpine , D. C. Lankin , S.‐N. Chen , and G. F. Pauli , “Natural Deep Eutectic Solvents: Properties, Applications, and Perspectives,” Journal of Natural Products 81 (2018): 679–690. 29513526 10.1021/acs.jnatprod.7b00945 PMC5913660

[8] A. Paiva , R. Craveiro , I. Aroso , M. Martins , R. L. Reis , and A. R. C. Duarte , “Natural Deep Eutectic Solvents – Solvents for the 21st Century,” ACS Sustainable Chemistry & Engineering 2 (2014): 1063–1071.

[9] A. P. Abbott , G. Capper , D. L. Davies , H. L. Munro , R. K. Rasheed , and V. Tambyrajah , “Preparation of Novel, Moisture‐Stable, Lewis‐Acidic Ionic Liquids Containing Quaternary Ammonium Salts with Functional Side Chains,” Chemical Communications (2001): 2010–2011. 12240264 10.1039/b106357j

[10] D. O. Abranches and J. A. P. Coutinho , “Everything You Wanted to Know about Deep Eutectic Solvents but Were Afraid to Be Told,” Annual Review of Chemical and Biomolecular Engineering 14 (2023): 141–163. 10.1146/annurev-chembioeng-101121-085323 36888992

[11] D. J. G. P. van Osch , C. H. J. T. Dietz , S. E. E. Warrag , and M. C. Kroon , “The Curious Case of Hydrophobic Deep Eutectic Solvents: A Story on the Discovery, Design, and Applications,” ACS Sustainable Chemistry & Engineering 8 (2020): 10591–10612.

[12] K. A. Omar and R. Sadeghi , “Physicochemical Properties of Deep Eutectic Solvents: A Review,” Journal of Molecular Liquids 360 (2022): 119524.

[13] Y. Chen and T. Mu , “Revisiting Greenness of Ionic Liquids and Deep Eutectic Solvents,” Green Chemical Engineering 2 (2021): 174–186.

[14] D. A. Alonso , A. Baeza , R. Chinchilla , G. Guillena , I. M. Pastor , and D. J. Ramón , “Deep Eutectic Solvents: The Organic Reaction Medium of the Century,” European Journal of Organic Chemistry (2016): 612–632.

[15] H. Tavakol and P. Shafieyoon , “Recent Advances and New Trends in the use of Deep Eutectic Solvents in Organic Synthesis and Other Applications,” Journal of Molecular Liquids 428 (2025): 127510.

[16] S. E. Hooshmand , R. Afshari , D. J. Ramón , and R. S. Varma , “Deep Eutectic Solvents: Cutting‐Edge Applications in Cross‐Coupling Reactions,” Green Chemistry 22 (2020): 3668–3692.

[17] L. Peng , Z. Hu , Q. Lu , Z. Tang , Y. Jiao , and X. Xu , “DESs: Green Solvents for Transition Metal Catalyzed Organic Reactions,” Chinese Chemical Letters 30 (2019): 2151–2156.

[18] J. García‐Álvarez , “Deep Eutectic Mixtures: Promising Sustainable Solvents for Metal‐Catalysed and Metal‐Mediated Organic Reactions,” European Journal of Inorganic Chemistry (2015): 5147–5157.

[19] A. E. Ünlü , A. Arıkaya , and S. Takaç , “Use of Deep Eutectic Solvents as Catalyst: A Mini‐Review,” Green Processing and Synthesis 8 (2019): 355–372.

[20] S. Khandelwal , Y. K. Tailor , and M. Kumar , “Deep Eutectic Solvents (DESs) as Eco‐Friendly and Sustainable Solvent/Catalyst Systems in Organic Transformations,” Journal of Molecular Liquids 215 (2016): 345–386.

[21] S. Pasricha , P. Gahlot , T. M. Rangarajan , et al., “Deep Eutectic Solvents (DESs): Emerging Viable Solvent Systems for Transition‐Metal‐Catalyzed Cross‐Coupling Reactions,” Journal of Molecular Liquids 426 (2025): 127287.

[22] F. G. Calvo‐Flores and C. Mingorance‐Sánchez , “Deep Eutectic Solvents and Multicomponent Reactions: Two Convergent Items to Green Chemistry Strategies,” ChemistryOpen 10 (2021): 815–829. 34402596 10.1002/open.202100137 PMC8369850

[23] C. O. Kappe , A. Stadler , and D. Dallinger , Microwaves in Organic and Medicinal Chemistry (Wiley‐VCH, 2012).

[24] R. J. Giguere , T. L. Bray , S. M. Duncan , and G. Majetich , “Application of Commercial Microwave Ovens to Organic Synthesis,” Tetrahedron Letters 27 (1986): 4945–4948.

[25] R. Gedye , F. Smith , K. Westaway , et al., “The use of Microwave Ovens for Rapid Organic Synthesis,” Tetrahedron Letters 27 (1986): 279–282.

[26] J. González‐Rivera , A. Mero , E. Husanu , et al., “Combining Acid‐Based Deep Eutectic Solvents and Microwave Irradiation for Improved Chestnut Shell Waste Valorization,” Green Chemistry 23 (2021): 10101–10115.

[27] C. Pelosi , J. Gonzalez‐Rivera , L. Bernazzani , M. R. Tiné , and C. Duce , “Optimized Preparation, Thermal Characterization and Microwave Absorption Properties of Deep Eutectic Solvents Made by Choline Chloride and Hydrated Salts of Alkali Earth Metals,” Journal of Molecular Liquids 371 (2023): 121104.

[28] J. González‐Rivera , E. Husanu , A. Mero , et al., “Insights into Microwave Heating Response and Thermal Decomposition Behavior of Deep Eutectic Solvents,” Journal of Molecular Liquids 300 (2020): 112357.

[29] M. Fu , H. Zhang , J. Bai , et al., “Application of Deep Eutectic Solvents with Modern Extraction Techniques for the Recovery of Natural Products: A Review,” ACS Food Science & Technology 5 (2025): 444–461.

[30] J. Osamede Airouyuwa , N. Sivapragasam , A. Ali Redha , and S. Maqsood , “Sustainable Green Extraction of Anthocyanins and Carotenoids Using Deep Eutectic Solvents (DES): A Review of Recent Developments,” Food Chemistry 448 (2024): 139061. 38537550 10.1016/j.foodchem.2024.139061

[31] T. Xiao , M. Hou , X. Guo , et al., “Recent Progress in Deep Eutectic Solvent (DES) Fractionation of Lignocellulosic Components : A Review,” Renewable and Sustainable Energy Reviews 192 (2024): 114243.

[32] Y. S. Wong , R. Yusoff , and G. C. Ngoh , “Phenolic Compounds Extraction by Assistive Technologies and Natural Deep Eutectic Solvents,” Reviews in Chemical Engineering 40 (2024): 229–246.

[33] R. Zou , X. Zhou , M. Qian , et al., “Advancements and Applications of Microwave‐Assisted Deep Eutectic Solvent (MW‐DES) Lignin Extraction: A Comprehensive Review,” Green Chemistry 26 (2024): 1153–1169.

[34] U. Patil , S. Shendage , and J. Nagarkar , “One‐Pot Synthesis of Nitriles From Aldehydes Catalyzed by Deep Eutectic Solvent,” Synthesis 45 (2013): 3295–3299.

[35] W.‐H. Zhang , M.‐N. Chen , Y. Hao , X. Jiang , X.‐L. Zhou , and Z.‐H. Zhang , “Choline Chloride and Lactic Acid: A Natural Deep Eutectic Solvent for One‐Pot Rapid Construction of Spiro[indoline‐3,4′‐Pyrazolo[3,4‐*b*]pyridines],” Journal of Molecular Liquids 278 (2019): 124–129.

[36] M. Zhang , P. Liu , Y.‐H. Liu , Z.‐R. Shang , H.‐C. Hu , and Z.‐H. Zhang , “Magnetically separa*b*le Graphene Oxide Anchored Sulfonic Acid: A Novel, Highly Efficient and Recyclable Catalyst for One‐Pot Synthesis of 3,6‐di(pyridin‐3‐yl)‐1H‐Pyrazolo[3,4‐b]pyridine‐5‐Carbonitriles in Deep Eutectic Solvent under Microwave Irradiation,” RSC Advances 6 (2016): 106160–106170.

[37] M. Zhang , Y.‐H. Liu , Z.‐R. Shang , H.‐C. Hu , and Z.‐H. Zhang , “Supported Molybdenum on Graphene Oxide/Fe3O4:An Efficient, Magnetically Separable Catalyst for One‐Pot Construction of Spiro‐Oxindole Dihydropyridines in Deep Eutectic Solvent under Microwave Irradiation,” Catalysis Communications 88 (2017): 39–44.

[38] B. B. F. Mirjalili , N. Jalili Bahabadi , and A. Bamoniri , “Triethanolamine–sodium Acetate as a Novel Deep Eutectic Solvent for Promotion of Tetrahydrodipyrazolopyridines Synthesis under Microwave Irradiation,” Journal of the Iranian Chemical Society 18 (2021): 2181–2187.

[39] M. Komar , T. G. Kraljević , I. Jerković , and M. Molnar , “Application of Deep Eutectic Solvents in the Synthesis of Substituted 2‐Mercaptoquinazolin‐4(3H)‐Ones: A Comparison of Selected Green Chemistry Methods,” Molecules 27 (2022): 558. 35056873 10.3390/molecules27020558 PMC8780518

[40] M. Komar , V. Rastija , D. Bešlo , and M. Molnar , “Synthesis of Quinazolin‐4(3H)‐Ones in Natural Deep Eutectic Solvents: Comparison of Various Synthetic Methods and Calculation of ADME Properties,” Journal of Molecular Structure 1304 (2024): 137725.

[41] C.‐T. Ma , P. Liu , W. Wu , and Z.‐H. Zhang , “Low meltin*g* Oxalic Acid/Proline Mixture as Dual Solvent/Catalyst for Efficient Synthesis of 13‐Aryl‐13 H ‐*b*enzo[g]benzothiazolo[2,3‐b]buinazoline‐5,4‐Diones under Microwave Irradiation,” Journal of Molecular Liquids 242 (2017): 606–611.

[42] U. Dasmahapatra , B. Choudhury , M. G. Ahmad , B. Maiti , and K. Chanda , “Dire*c*t Access to 5,6‐Dihydropyrido[2′,1′: 2,3]pyrido‐Fused Imidazo [4,5‐c]quinolines via Consecutive C–N and C–C Bond Formation in Deep Eutectic Solvent under Microwave Irradiation,” Synthesis 57 (2025): 1007–1014.

[43] E. Brambilla , A. Gritti , V. Pirovano , et al., “Acidic Deep Eutectic Solvents as Active Media for Sustainable Synthesis of Biindoles Starting From 2,2′‐Diaminotolanes and Aldehydes,” European Journal of Organic Chemistry 26 (2023): e202300204.

[44] S. Tabasso , R. Moro , E. Calcio Gaudino , C. Bruschetta , and G. Cravotto , “Sustainable Microwave‐Assisted Routes to *N* ‐Substituted Pyrrolidones Using Natural Deep Eutectic Solvents as Non‐Innocent Solvents,” ACS Sustainable Chemistry & Engineering 12 (2024): 13810–13815.

[45] C. Teja , A. Garg , G. K. Rohith , H. Roshini , S. Jena , and F. R. Nawaz Khan , "Diversity Oriented Synthesis of Oxygen‐Heterocycles, Warfarin Analogs Utilizing Microwave‐Assisted Dimethyl Urea‐Based Deep Eutectic Solvents," Polycyclic Aromatic Compounds 42 (2022): 4769–4779.

[46] L. Xu , W.‐H. Zhang , Z.‐S. Cui , and Z.‐H. Zhang , “Choline Chloride/Glycerol Promoted Synthesis of 3,3‐Disubstituted Indol‐ 2‐Ones,” Current Organocatalysis 8 (2021): 249–257.

[47] F. Curti , M. Tiecco , V. Pirovano , et al., “p‐TSA‐Based DESs as Active Green Solvents for Microwave Enhanced Cyclization of 2‐Alkynyl‐(hetero)‐Arylcarboxylates: An Alternative Access to 6‐Substituted 3,4‐Fused 2‐Pyranones,” European Journal of Organic Chemistry (2019): 1904–1914.

[48] Y. Riadi , O. Ouerghi , M. H. Geesi , A. Kaiba , E. H. Anouar , and P. Guionneau , “Efficient Novel Eutectic‐Mixture‐Mediated Synthesis of Benzoxazole‐Linked Pyrrolidin‐2‐One Heterocycles,” Journal of Molecular Liquids 323 (2021): 115011.

[49] P. T. Pham , H. T. Nguyen , T. T. Nguyen , et al., “Rapid and Simple Microwave‐Assisted Synthesis of Benzoxazoles Catalyzed by [CholineCl][Oxalic Acid],” Catalysts 12 (2022): 1394.

[50] A. Castro , I. M. G. Andrade , M. C. Coelho , et al., “Multicomponent Synthesis of Spiro 1,3,4‐Thiadiazolines with Anticancer Activity by Using Deep Eutectic Solvent under Microwave Irradiation,” Journal of Heterocyclic Chemistry 60 (2023): 392–405.

[51] D. P. da Costa , A. C. de Castro , G. A. da Silva , et al., “Microwave‐Assisted Synthesis and Antimicrobial Activity of Novel Spiro 1,3,4‐Thiadiazolines From Isatin Derivatives,” Journal of Heterocyclic Chemistry 58 (2021): 766–776.

[52] M. Shaikh , M. Shaikh , D. Wagare , A. Ahmed Sheikh , and S. Sultan Kasim , “Deep Eutectic Solvent (DES) Mediated Multicomponent Synthesis of 4‐ Thiazolidinone‐5‐Carboxylic Acid: A Green Chemistry Approach,” Current Catalysis 11 (2022): 65–70.

[53] V. Bušić , S. Roca , D. Vikić‐Topić , et al., “Eco‐Friendly Quaternization of Nicotinamide and 2‐Bromoacetophenones in Deep Eutectic Solvents. Antifungal Activity of the Products,” Environmental Chemistry Letters 18 (2020): 889–894.

[54] V. Bušić , M. Molnar , V. Tomičić , D. Božanović , I. Jerković , and D. Gašo‐Sokač , “Choline Chloride‐Based Deep Eutectic Solvents as Green Effective Medium for Quaternization Reactions,” Molecules 27 (2022): 7429. 36364264 10.3390/molecules27217429 PMC9655353

[55] V. Bušić , K. Vrandečić , T. Siber , S. Roca , V. Tomičić , and D. Gašo Sokač , “Novel Synthetic Routes to Quaternary Pyridinium Salts and Their Antifungal Activity,” Croatica Chemica Acta 95 (2022): 31–38.

[56] C. Chen , M. Lv , H. Hu , et al., “5‐Hydroxymethylfurfural and Its Downstream Chemicals: A Review of Catalytic Routes,” Advanced Materials 36 (2024): 2311464. 10.1002/adma.202311464 38808666

[57] Q. Li , K. Sun , Y. Shao , et al., “Coordination of Acidic Deep Eutectic Solvent–Chromium Trichloride Catalytic System for Efficient Synthesis of Fructose to 5‐Hydroxymethylfurfual,” Industrial & Engineering Chemistry Research 59 (2020): 17554–17563.

[58] E. S. Morais , M. G. Freire , C. S. R. Freire , and A. J. D. Silvestre , “Improved Production of 5‐Hydroxymethylfurfural in Acidic Deep Eutectic Solvents Using Microwave‐Assisted Reactions,” International Journal of Molecular Sciences 23 (2022): 1959. 35216072 10.3390/ijms23041959 PMC8875992

[59] G. A. D. Castro , S. A. Fernandes , R. de C. S. de Sousa , and M. M. Pereira , “Green Synthesis of 5‐Hydroxymethylfurfural and 5‐Acetoxymethylfurfural Using a Deep Eutectic Solvent in a Biphasic System Assisted by Microwaves,” Reaction Chemistry & Engineering 8 (2023): 1324–1333.

[60] İ. Aslanlı and M. Sert , “One Pot Conversion of Fructose to Alkyl Levulinates Catalyzed by Deep Eutectic Solvents in Microwave Reactor,” Renewable Energy 244 (2025): 122698.

[61] K. M. Taylor , Z. E. Taylor , and S. T. Handy , “Rapid Synthesis of Aurones under Mild Conditions Using a Combination of Microwaves and Deep Eutectic Solvents,” Tetrahedron Letters 58 (2017): 240–241.

[62] M.‐A. Karadendrou , I. Kostopoulou , V. Kakokefalou , A. Tzani , and A. Detsi , “L‐Proline‐Based Natural Deep Eutectic Solvents as Efficient Solvents and *Catalysts* for the Ultrasound‐Assisted Synthesis of Aurones via Knoevenagel Condensation, Catalysts 12 (2022): 249.

[63] S. Hesse , J. Hertzog , and S. Rup‐Jacques , “L‐Proline‐Based DES in Knoevenagel Synthesis of Arylidene Rhodanines, Thiazolidine‐2,4‐Diones, and Barbituric Derivatives,” Journal of Heterocyclic Chemistry 61 (2024): 1015–1023.

[64] G. Colombo Dugoni , A. Sacchetti , and A. Mele , “Deep Eutectic Solvent as Solvent and Catalyst: One‐Pot Synthesis of 1,3‐Dinitropropanes *via* Tandem Henry Reaction/Michael Addition,” Organic & Biomolecular Chemistry 18 (2020): 8395–8401. 32845266 10.1039/d0ob01516d

[65] M. Shaibuna , K. Hiba , and K. Sreekumar , “Deep Eutectic Solvent for the Synthesis of (E)‐ Nitroalkene via Microwave Assisted Henry Reaction,” Current Research in Green and Sustainable Chemistry 4 (2021): 100187.

[66] E. Brambilla , A. Bortolla , V. Pirovano , A. Caselli , M. Tiecco , and G. Abbiati , “Silver‐Catalysed A ^3^ ‐Coupling Reactions in Phenylacetic Acid/Alkylamine *N* ‐Oxide Eutectic Mixture under Dielectric Heating: An Alternative Approach to Propargylamines,” Applied Organometallic Chemistry 36 (2022): e6669.

[67] M. Molnar , T. Gazivoda Kraljević , V. Pavić , V. Rastija , and M. Komar , “Environmentally Friendly Approach to the Synthesis of 3‐[Benzylideneamino]‐2‐Methylquinazolin‐4(3H)‐One Derivatives and Calculation of Their Toxicity,” Chemistry & Biodiversity 20 (2023): e202300575. 37417922 10.1002/cbdv.202300575

[68] A. K. Drabczyk , D. Kułaga , P. Zaręba , et al., “Eco‐Friendly Synthesis of New Olanzapine Derivatives and Evaluation of Their Anticancer Potential,” RSC Advances 13 (2023): 20467–20476. 37435368 10.1039/d3ra03926a PMC10331126

[69] C. Salami , L. Boudesocque‐Delaye , P.‐O. Delaye , and E. Thiery , “Simple and Rapid Microwave‐Assisted Suzuki–Miyaura Cross‐Coupling in Betaine/Glycerol Natural Eutectic Solvent,” ChemistryOpen 00 (2025): e202500138. 10.1002/open.202500138 PMC12598815 40528533

[70] C. R. McElroy , A. Constantinou , L. C. Jones , L. Summerton , and J. H. Clark , “Towards a Holistic Approach to Metrics for the 21st Century Pharmaceutical Industry,” Green Chemistry 17 (2015): 3111–3121.

[71] A. DeVierno Kreuder , T. House‐Knight , J. Whitford , et al., “A Method for Assessing Greener Alternatives between Chemical Products Following the 12 Principles of Green Chemistry,” ACS Sustainable Chemistry & Engineering 5 (2017): 2927–2935.

